# Review: Psychological & Behavioural Treatments of Nonepileptic Seizures in Children and Adolescents

**DOI:** 10.1192/bjo.2021.791

**Published:** 2021-06-18

**Authors:** Hemma Velani, Julia Gledhill

**Affiliations:** Central and North West London NHS Foundation Trust

## Abstract

**Aims:**

To systematically review Psychological and Behavioural treatments on NES in children and adolescents by reviewing the current literature.

**Background:**

Non-epileptic seizures (NES) are associated with a high level of functional impairment for young people and their families. However, there are no UK guidelines for the management of NES in children and adolescents or adults. Though information from the limited studies in adults may be useful, the findings may not be generalizable to children and adolescents. To date, we are unaware of any published systematic review on this topic in children and adolescents.

**Method:**

A systematic search of relevant electronic databases was conducted. Any study investigating the effectiveness of psychological and behavioural treatments on NES, in Children and Adolescents was included.

**Result:**

Fifteen studies were identified, but only six studies had the primary aim of evaluating an intervention, and only one used a control group. The rest were observational studies that examined retrospective case notes.

CBT and psychoeducation were identified as the most common interventions. Eleven out of the fifteen studies used multiple treatments, four looked at one treatment only, three of these CBT and one was a natural history study.

Where individual therapy was provided, a common focus was management of anxiety, usually delivered in a flexible way, adapted to individual needs. Despite being identified as important in the literature, only one study demonstrated care that involved collaboration between physical and mental health teams.

**Conclusion:**

It's difficult to conclude from this review that one treatment approach is superior to another. The findings of this review offer some insight into current practise and may help to inform future research in this area. CBT and psychoeducation with a focus on anxiety are frequently included in interventions for NES in young people, and further evaluation of these treatment modalities could be a helpful next step.

